# 3T-MRH1 spectroscopy for quantification of myocardial steatosis: relationship to metabolic profile and global myocardial function

**DOI:** 10.1186/1532-429X-15-S1-W16

**Published:** 2013-01-30

**Authors:** R Noureldin, R Ouwerkerk, M Warren, S Sable, M Skarulis, A Gharib, RI Pettigrew

**Affiliations:** 1 NIDDK, Bethesda, MD, USA

## Background

The major adverse consequence of obesity and metabolic syndrome is cardiovascular disease and is attributed to myocardial steatosis. CMR proton spectroscopy has the potential for quantification of amount of fat deposited in the heart.

## Methods

A research protocol was applied on 90 subjects, using a Siemens Verio 70 cm bore 3T-MRI scanner. All subjects were not known to have cardiac disease. MR spectroscopy was performed with water suppressed ECG gated point resolved spectroscopy (PRESS), TR/TE =1R-R/30ms. PRESS voxel was located in the septum at isovolumic phase of diastole. Short axis and 4-chamber Steady State Free Precession (SSFP) cine images were obtained to prescribe the PRESS voxel location, and saturation slabs across subcutaneous and pericardial fat were used.

A breathing navigator was set on the dome of the diaphragm on a free breathing scout to reduce the breathing effects. B0 shimming parameters were optimized with a (breath-hold) rapid B0 mapping method.

Fat content was quantified with Amares/MRUI and related to water in unsuppressed spectra. SSFP images were used for evaluation of global myocardial function (ejection fraction, end diastolic volume, end systolic volume, stroke volume and cardiac output). Variables were indexed to BSA.

All subjects had same day metabolic profile evaluation, included insulin sensitivity, fasting glucose level, resting energy expenditure, maximum VO2, regional fat percentage and waist circumference. Lipid profile including serum cholesterol, HDL, LDL and serum triglycerides was obtained within one month of the scan.

## Results

Seventy-seven MRS scans were analyzable, mean age 51±12 years, 46% were men, and 69% were white. BMI ranged from 19.4 to 54.8 and 36.4% had metabolic syndrome. Mean EF, EDVI, ESVI and LVMI were 62±6%, 60±15,23±7 and 54±11 respectively. Myocardial fat content ranged from 0.03 to 3.94 %, it showed mild positive correlation with BMI, waist circumference and triglycerides (r= 0.26, 0.3 and 0.23 respectively, p <0.05). There was a trend to positive correlation with free fatty acids (r= 0.22, P=0.07), however, end diastolic volume and stroke volume indices showed negative correlation (r= -0.23 and -0.25, P < 0.05).

## Conclusions

Myocardial 1HMR Spectroscopy is a promising tool for quantification of ectopic fat deposition in the myocardium. High triglycerides in the body is highly associated with fat accumulation around the heart. In population with no cardiac disease, higher myocardial fat percentage is associated with lower EDVI and ESVI.

